# Article Patellar aneurysmal bone cyst in children: a case report

**Published:** 2012-07-18

**Authors:** Mounir Arroud, Karima Atarraf, Lamiae Chater, My Abderrahmane Afifi

**Affiliations:** 1 Pediatric orthopedic department, Hassan II Hospital, Fes, Morocco

**Keywords:** Patellar, aneurysm, cyst, bone, children

## Abstract

An aneurysmal bone cyst is rare in the patella. We report the case of a 9 year-old boy who presented with chronic pain and no previous trauma history. Treatment included curettage of the cyst and filling with a iliac bone graft. Ten months after surgery, the knee was mobile and painless and graft incorporation was good.

## Introduction

An aneurysmal bone cyst is a benign aggressive bone lesion first described by Jaffe and Lichtenstein in 1942 [1]. It is typically an expansile osteolytic lesion consisting of blood-filled spaces and channels that are divided by connective tissue septa, which may contain osteoid tissue and osteoclastlike giant cells [2]. A neurysmal bone cysts comprise less that 1% of all primary bone tumors. There are few reported cases of aneurysmal bone cyst of the patella [3–11]. The most common extrapatellar site is the distal femur and proximal tibia, followed by the spine and pelvis [12]. We report a particularly young patient with an aneurysmal bone cyst of the patella treated by curettage and iliac grafting with good result.

## Patient and observation

A 9-year old boy presented to the pediatric department with chronic right knee pain and swelling which had increased during the previous 3 months. There was no history of recent skin or other infections, trauma, fever, or constitutional symptoms or signs. His medical history and family history were non contributory. A physical examination revealed tenderness and swelling over the patella. The patient had a full, active range of knee movement.

**Figure 1:**
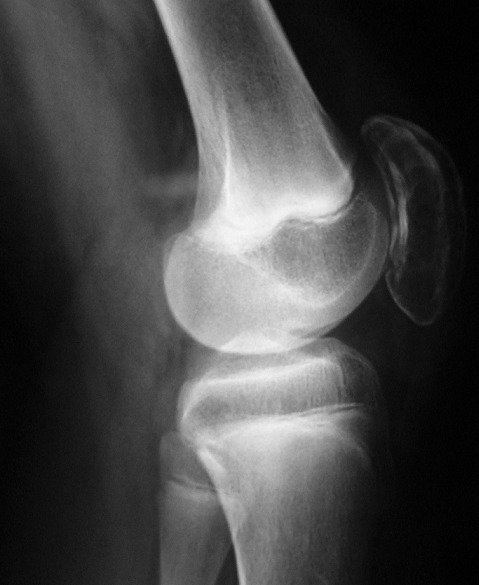
Radiography of the right knee showing an osteolytic lesion involving the hole patella

**Figure 2:**
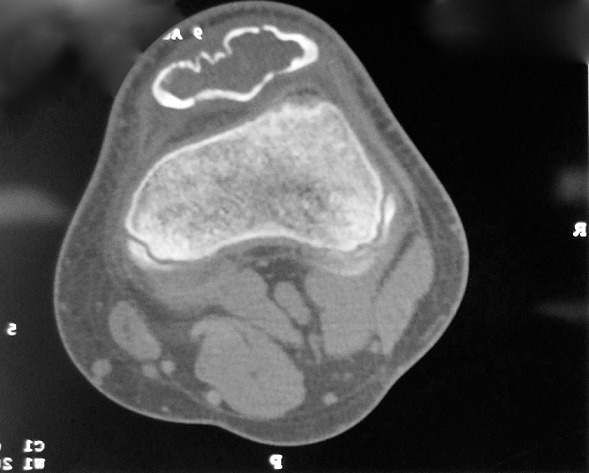
Sagittal CT scan image showing a multi-loculated lesion in the patella

Radiographs of the right knee showed an osteolytic lesion involving the hole patella. Endosteal scalloping and cortical thinning with mild expansion and a multi-loculated appearance was observed (Figure 1). Sagittal CT scan images showed a multi-loculated lesion in the patella (Figure 2).

**Figure 3:**
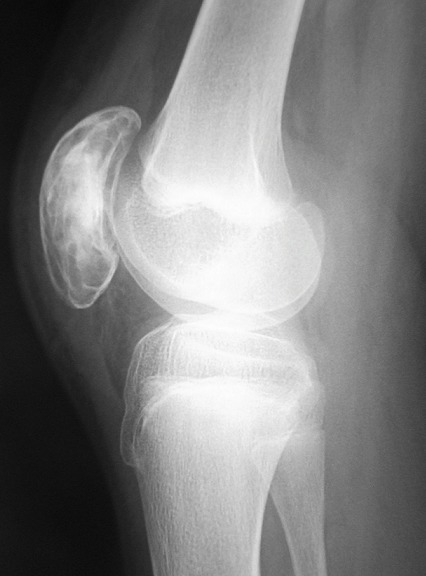
Radiography of the right knee showing the iliac graft packing of the patella

An open bone biopsy of the patella with intraoperative frozen section was performed. The soft tissues appeared normal, but a thin anterior cortex of the patella was seen and punctured with an elevator. After removal of a large window of thin cortical bone, a large well-defined cavity filled with serosanguinous fluid was found. A rim of tissue that lined the cavity and curettings from within the cavity were sent for histologic analysis. The bony rim was sclerotic and intact. The posterior wall of the cavity was cartilaginous, but the patellar cartilage revealed no obvious violation of the joint surface. There was no evidence of malignancy. Intraoperative Gram stain and cultures were negative. The lesion was diagnosed as a bone cyst pending histologic analysis. Based on these intraoperative findings, the lesion was completely curetted. The periphery of the cavity was cauterized and then burred. The defect was packed with autologous iliac graft (Figure 3).

Histopathologic examination of the biopsy material revealed empty spaces outlined by thin septa made of spindle cells, randomly scattered benign giant cells, and capillaries in a collagenous matrix. The septa were made of fibrous connective tissue containing fine strands of immature woven bone. Fiber osteoid was observed in some septa. There were no mitoses or atypical cells. These histologic findings were consistent with an aneurysmal bone cyst.

Ten months after intralesional curettage and bone grafting, our patient returned to full activities without pain. He has regained full range of motion of his knee.

## Discussion

Patellar tumors are uncommon, but almost 75% of these diagnosed in children and adults are benign [13]. Giant cell tumor and chondroblastoma are the most common benign patellar neoplasms whereas osteosarcoma is the most frequent primary malignancy [13]. Aneurysmal bone cysts account for less than 1% of primary bone tumours and have a predilection for the metaphysis of the long bones of the leg. Only 1% of all aneurysmal bone cysts occur in the patella.

Most patients with primary patellar tumours are young and active, and give a history of knee pain, swelling, and related trauma.

Radiologically aneurysmal bone cysts appear as an eccentric or central osteolytic lesion with cortical expansion, giving a ‘blown-out’ appearance with extension into soft tissues. Trabeculae are rude at the periphery of the lesion but become delicate toward the center [14]. Osteolytic lesions are surrounded by bony septa and the surface of the intra-osseous border shows periosteal and new bone formation. Benign chondroblastoma and bony giant cell tumor must be considered in the differential diagnosis of aneurysmal bone cyst since these tumors usually show the same clinical and plain X-ray features.

CT scan and MRI display a better evaluation of internal architecture of the tumor. They demonstrate the presence of mineralization, expansion of the bone and changes in the cortex. They can also evaluate the extraosseous extent of the lesion. However, it is well known that neither CT scan nor MRI adds anything to plain radiographs in establishing the diagnosis of aneurysmal bone cyst [15].

Intralesional curettage and bone grafting are the preferred treatment for benign patella tumors without articular involvement. Total patellectomy or patellar prosthesis is recommended for aggressive lesions that disrupt the patellar articular surface [7, 9]. This technique is rarely used in children. Recurrence rates after curettage vary widely from 9% to 71%, with lesions of the humerus and femur most common [16, 17]. Some authors have reported higher recurrence rates with aneurysmal bone cysts in children younger than 15 years [18]. The average time between surgery and initial recurrence was 7.6 months [17]. Repeated extended curettage and bone graft are recommended in the event of recurrence

## Conclusion

Aneurysmal bone cysts is benign tumor that may mimic other tubular bone's benign tumors. Its occurrence in the patella in children is highly exceptional. The classic radiographic appearances are not specific. Consequently, the diagnosis is most often made by histopathological assessment. The preferred treatment is intralesional curettage and bone grafting
